# Supplementary material for: Directional integration and pathway enrichment analysis for multi-omics data
Source: Nat Commun. 2024 Jul 7;15:5690. doi: 10.1038/s41467-024-49986-4 (PMC11227559; doi:10.1038/s41467-024-49986-4)
Supplement: Supplementary file 1 — Supplementary Information [file 41467_2024_49986_MOESM1_ESM.pdf]

Directional integration and pathway enrichment analysis for multi-omics data

SUPPLEMENTARY INFORMATION

Mykhaylo Slobodyanyuk[*], Alexander T. Bahcheli[*], Zoe P. Klein, Masroor Bayati, Lisa J. Strug, Jüri Reimand[@]

* − these authors contributed equally
@ − correspondence: juri.reimand@utoronto.ca

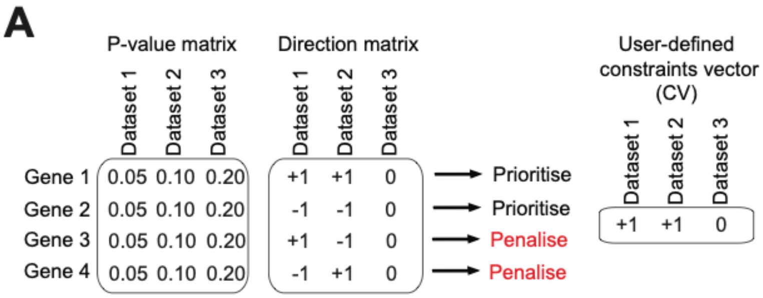

B

$$d_i = o_i \, e_i$$

$$X_{\mathrm{DPM}} = -2\left(-\left|\sum_{i=1}^{j} d_i \ln(P_i)\right| + \sum_{i=j+1}^{k} \ln(P_i)\right) \qquad P'_{\mathrm{DPM}} = 1 - \chi^2\left(\frac{X_{\mathrm{DPM}}}{c}, k'\right)$$

Gene 1

$$d = [+1, +1, 0]\,[+1, +1, 0] = [+1, +1, 0]$$

$$X_{\mathrm{DPM}} = -2(-|(+1)\ln(0.05) + (+1)\ln(0.10)| + \ln(0.20))$$

$$X_{\mathrm{DPM}} = 13.81$$

$$P'_{\mathrm{DPM}} = 1 - \chi^2\left(\frac{13.81}{1}, 6\right) = 0.03$$

Gene 2

$$d = [-1, -1, 0]\,[+1, +1, 0] = [-1, -1, 0]$$

$$X_{\mathrm{DPM}} = -2(-|(-1)\ln(0.05) + (-1)\ln(0.10)| + \ln(0.20))$$

$$X_{\mathrm{DPM}} = 13.81$$

$$P'_{\mathrm{DPM}} = 1 - \chi^2\left(\frac{13.81}{1}, 6\right) = 0.03$$

Gene 3

$$d = [+1, -1, 0]\,[+1, +1, 0] = [+1, -1, 0]$$

$$X_{\mathrm{DPM}} = -2(-|(+1)\ln(0.05) + (-1)\ln(0.10)| + \ln(0.20))$$

$$X_{\mathrm{DPM}} = 4.61$$

$$P'_{\mathrm{DPM}} = 1 - \chi^2\left(\frac{4.61}{1}, 6\right) = 0.60$$

Gene 4

$$d = [-1, +1, 0]\,[+1, +1, 0] = [-1, +1, 0]$$

$$X_{\mathrm{DPM}} = -2(-|(-1)\ln(0.05) + (+1)\ln(0.10)| + \ln(0.20))$$

$$X_{\mathrm{DPM}} = 4.61$$

$$P'_{\mathrm{DPM}} = 1 - \chi^2\left(\frac{4.61}{1}, 6\right) = 0.60$$

Supplementary Figure 1. Example of directional P-value merging (DPM) in three input omics datasets. (A) Overview of directional analysis. Two genes with identical P-values and different directional changes are analysed from input Datasets 1-3. Three inputs are required: (i) P-values of Genes 1-4 from Datasets 1-3 derived from upstream analyses; (ii) directional changes of Genes 1-4 from Datasets 1-3, such as fold-change values, that are simplified in DPM as positive (+1) or negative (−1) unit values or as zero (0) if no directions are defined; and (iii) the user-defined constraints vector (CV) that shows how the input datasets are expected to interact directionally. In this example, Dataset 3 contains no directional information, while Dataset 1 and Dataset 2 have direct directional relationship indicated as [+1, +1, 0]. **(B)** Example of prioritising and penalising genes using directional information. Gene 3 and Gene 4 are penalised due to directional inconsistencies (red) while Gene 1 and Gene 2 are prioritised. The direction term d_i is acquired by element-wise multiplication of each gene's observed direction (o_i) and its expected direction from the CV (e_i). To evaluate the significance of directionally merged P-values, χ^2 values are acquired separately for directional datasets (Dataset 1, Dataset 2) and for the non-directional Dataset 3 and combined as a sum. Gene 1 and Gene 2 are directionally consistent and the resulting merged P-values from DPM are significant. In contrast, Gene 3 and Gene 4 are penalised due to observed directional inconsistencies relative to the CV and therefore these genes have non-significant merged P-values.

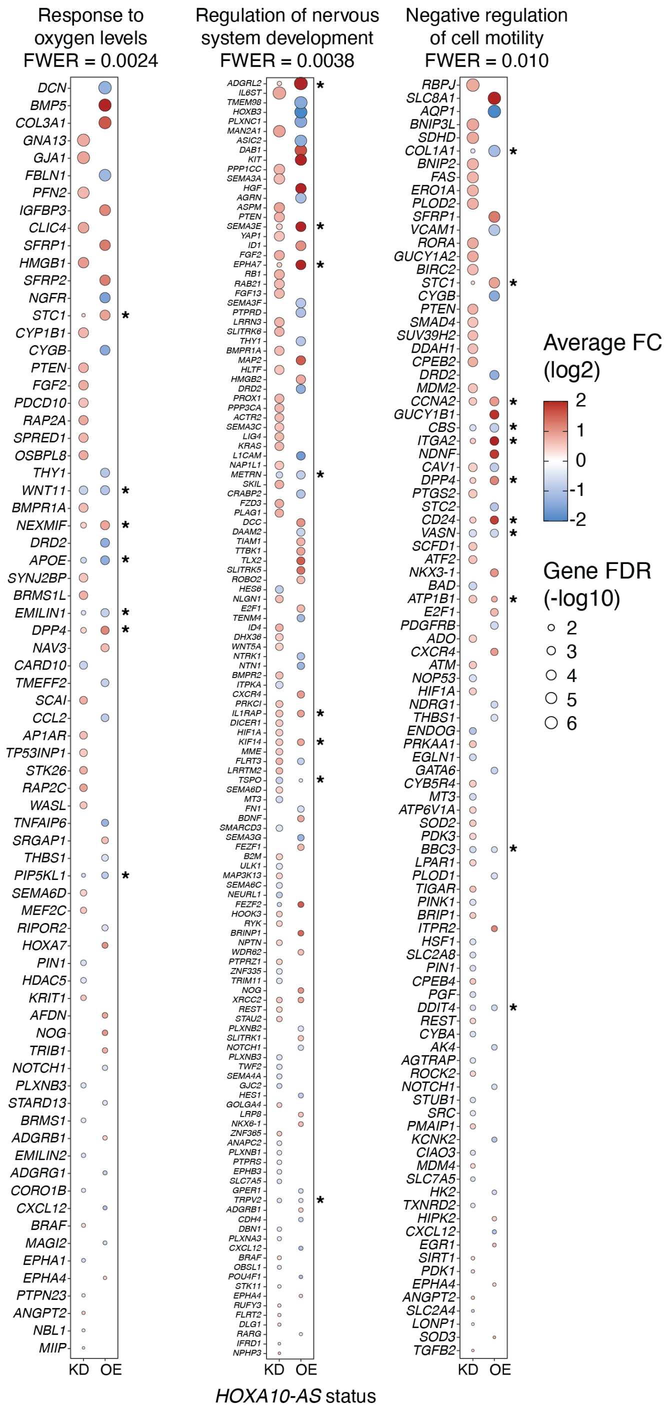

Supplementary Figure 2. Examples of directionally penalised pathways regulated by *HOXA10-AS* knockdown and overexpression. Directionally penalised pathways identified from transcriptomics data of *HOXA10-AS* knockdown (KD) and overexpression (OE) in glioma cells. DPM was used to prioritise genes with opposite changes in KD and OE using the constraints vector [KD = +1; OE = −1]. Dot plots show FDR and fold-change (FC) values of significant genes in representative pathways. FDR values and FC values are from the original study (Isaev et al 2021) and were originally derived using the EdgeR software and the TREAT statistical test. Gene P-values shown were adjusted for multiple testing. Asterisks indicate genes with inconsistent directions. While these processes are important in glioma, this analysis suggests that these may not be directly regulated by *HOXA10-AS* expression.

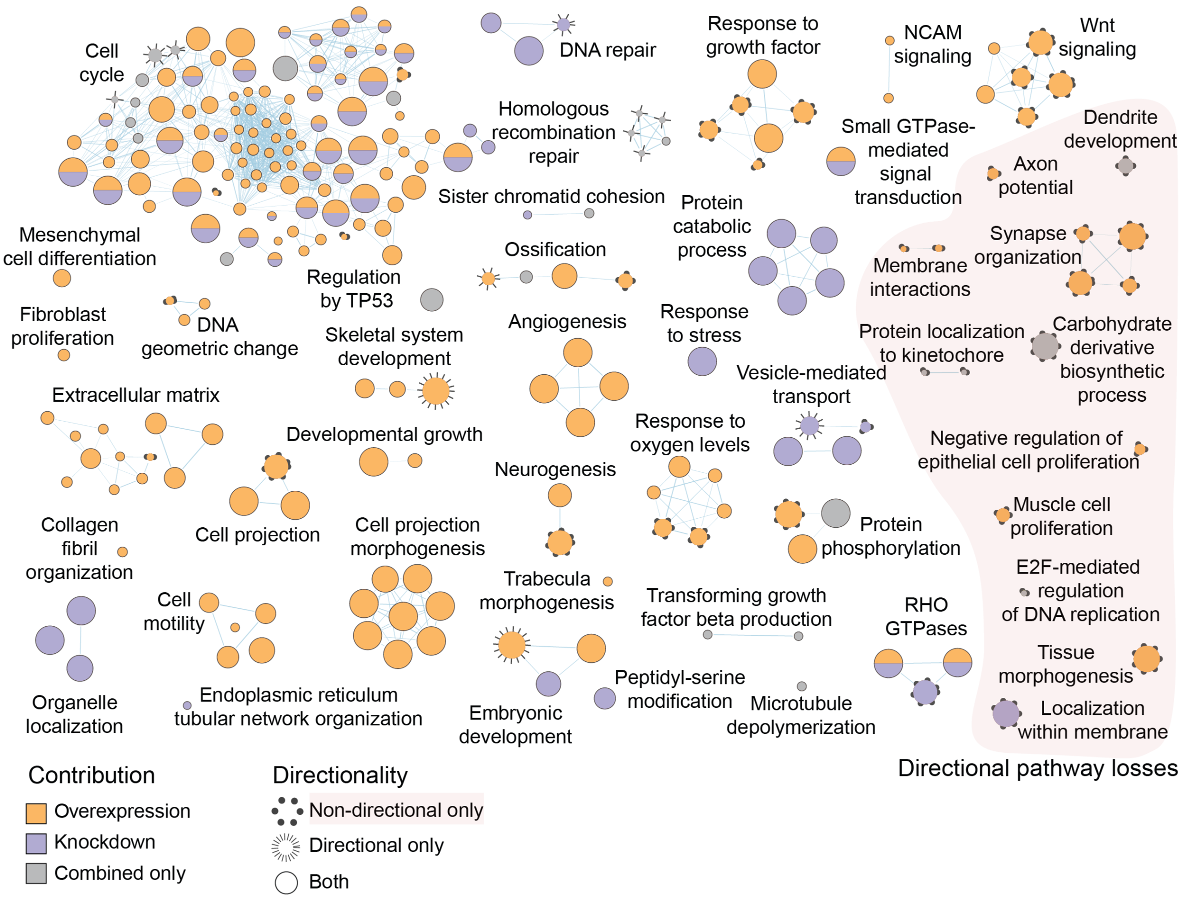

Supplementary Figure 3. Directional integration of genes and pathways jointly upregulated or downregulated in *HOXA10-AS* knockdown and overexpression experiments. Consistent fold-change (FC) directions for gene prioritisation were encoded in the constraints vector (CV) as [KD = +1, OE = +1]. Enriched pathways and processes from *HOXA10-AS* knockdown (KD) and overexpression (OE) experiments in glioma cells visualised as an enrichment map (ActivePathways, FWER < 0.05). The network shows pathways as nodes that are connected by edges and grouped into subnetworks if the corresponding pathways share many genes. Node color indicates the dataset contribution (KD, OE, both, or combined-only), and node sizes reflect the number of genes per pathway. Node outline indicates whether the pathways were identified using DPM alone (*i.e.*, the directional information helped prioritise pathway genes; spiky edges), the non-directional Brown method alone (*i.e.*, the directional information penalised pathway genes with inconsistent fold-changes; dotted edges), or using both approaches (*i.e.*, most pathway genes had consistent directional information; solid edges). Pink background highlights the major functional themes that were lost in the directional analysis.

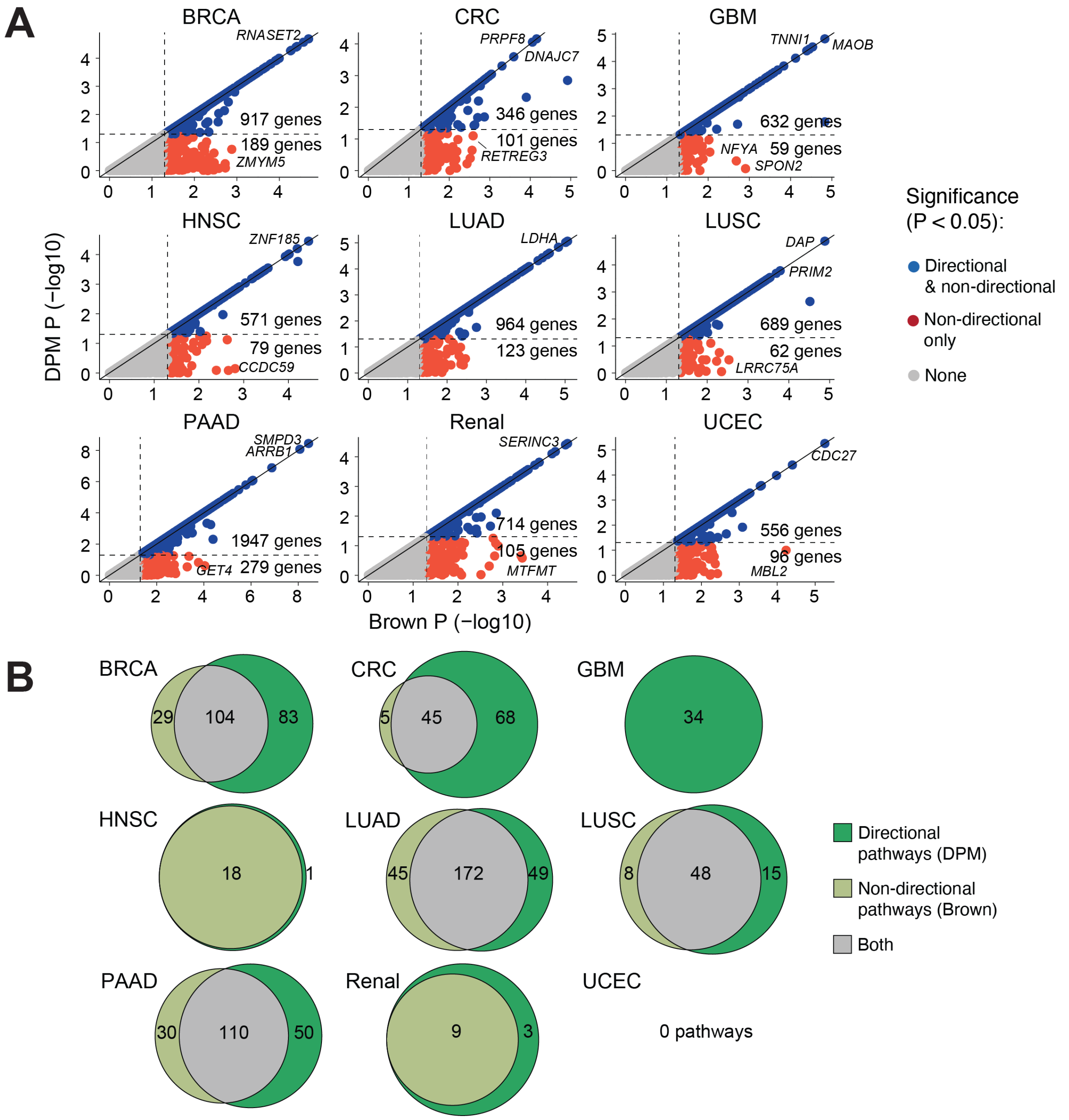

Supplementary Figure 4. Integrating transcriptomic and proteomic datasets with cancer patient survival in 10 cancer types to find prognostic biomarkers and enriched pathways. We used DPM to prioritise genes whose mRNA and protein levels associated consistently with better or worse prognosis of cancer patients using the constraints vector (CV) as [mRNA = +1, protein = +1]. Gene P-values and directional values were derived from Cox proportional-hazards models with protein or mRNA levels and clinical covariates (patient age, sex, and tumor stage) as predictors of overall survival (OS). We analysed each of 10 cancer types from the CPTAC and TCGA projects. Each gene was modelled twice: once with mRNA level and once with protein level as predictor of OS. Directional values were defined as signs of log-transformed hazard ratio (HR) values. **(A)** Directionally prioritised and penalised genes for 10 cancer types. Scatter plots show directionally merged P-values (DPM, Y-axis) and non-directional P-values (Brown; X-axis). Significant genes from DPM are shown in blue ($P < 0.05$). Gene P-values shown were not adjusted for multiple testing. **(B)** Pathway-level integration of OS with transcriptomics and proteomics data with and without directional information ($FDR < 0.05$). Venn diagrams show the numbers of significant pathways from directional (DPM) and non-directional analyses (Brown).

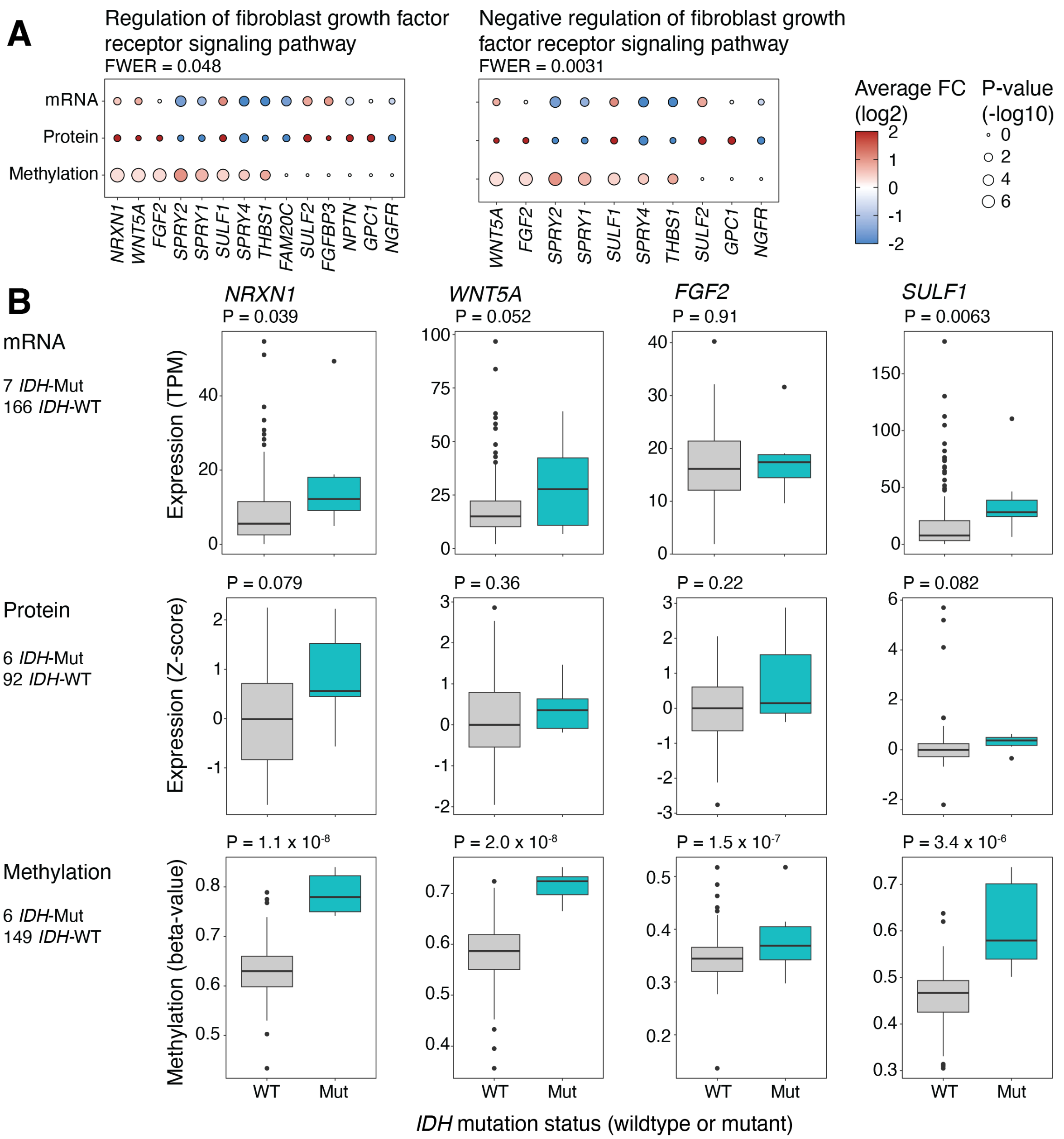

Supplementary Figure 5. Directional analysis of fibroblast growth factor receptor (FGFR) pathway in *IDH*-mutant gliomas. (A) Two FGFR pathways were directionally penalised in the directional integration of promoter DNA methylation with mRNA and protein expression. Constraints vector (CV) was defined to prioritise direct associations of mRNA and protein expression and inverse association with methylation [methylation = +1, mRNA = −1, protein = −1]. Dot plots show significant genes in two pathways identified in the non-directional analysis. **(B)** Four genes showing directional conflicts in which higher promoter methylation co-occurred with higher mRNA and protein expression in *IDH*-mutant *vs.* *IDH*-wildtype gliomas. Box plots show mRNA expression (top), protein expression (middle), and promoter methylation (bottom). Directional inconsistencies may be apparent as genetic alterations and deregulation of FGFR genes have been found in a subset of *IDH*-wildtype gliomas, while this analysis focuses on the *IDH*-mutant subtype. Box plots span interquartile range (IQR; 25-75%) where median values are shown as lines and whiskers show 1.5x of IQR. Unadjusted P-values from Mann-Whitney U-tests are shown.

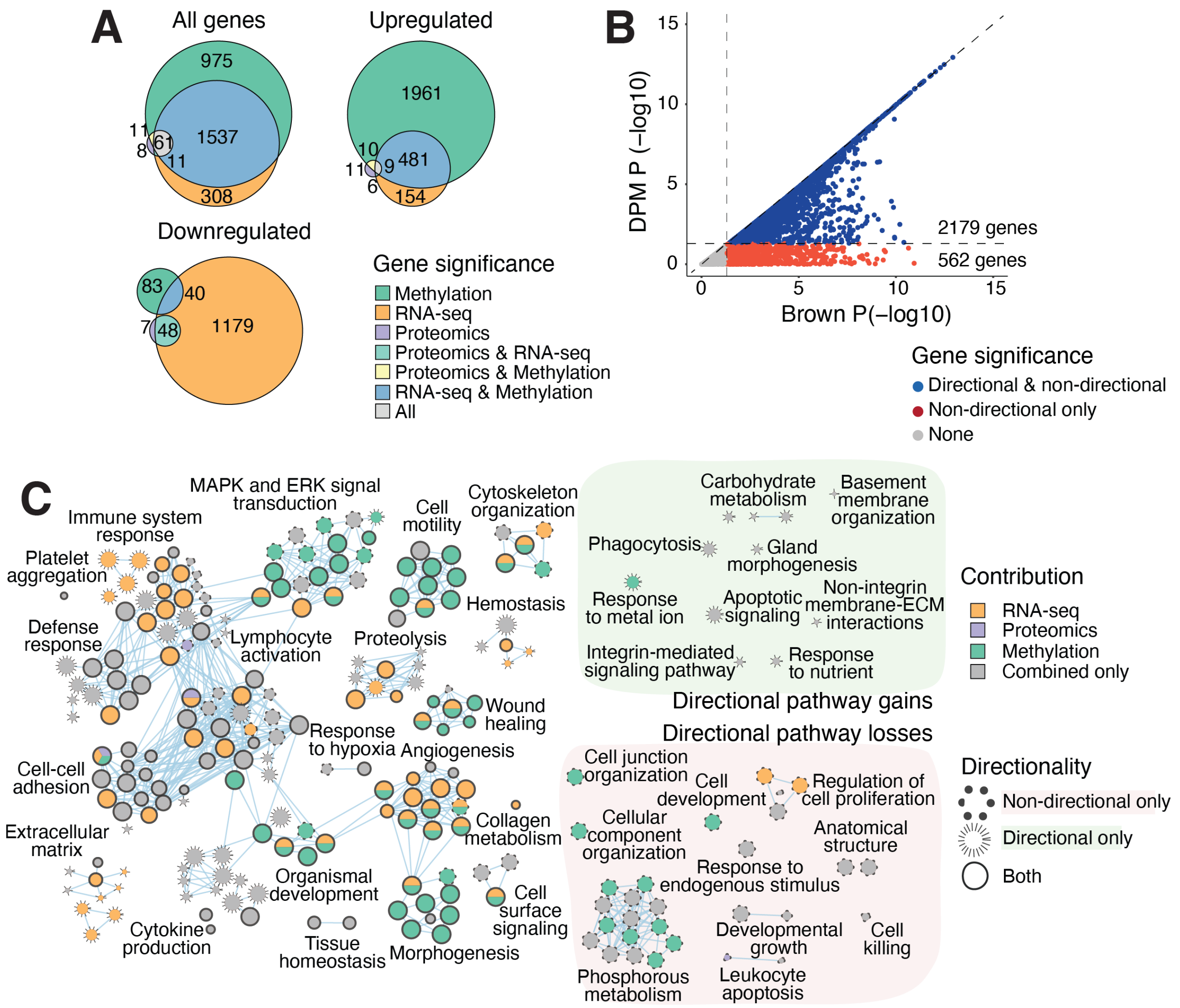

Supplementary Figure 6. Validating the integrative analysis of *IDH*-mutant gliomas in an independent dataset of cancer samples. We compared *IDH*-mutant and *IDH*-wildtype gliomas by integrating differential mRNA and protein expression and promoter DNA methylation using DPM. Multi-omics data for an independent set of gliomas were derived from the GLASS project and the study by Oh *et al*. (2022). The constraints vector (CV) defined directional associations between the three input datasets: mRNA and protein levels associated directly while mRNA and protein levels associated inversely with DNA promoter methylation, a repressive regulatory mechanism (CV = [methylation = +1, mRNA = −1, protein = −1]). **(A)** Venn diagrams show significant genes found separately in the three datasets (FDR < 0.1). Downregulated genes (bottom left) show reduced mRNA and protein expression and increased promoter methylation while upregulated genes show decreased promoter methylation and increased mRNA and protein expression (top right). **(B)** Scatter plot of directionally merged P-values of genes (DPM, Y-axis) and non-directional P-values of genes (Brown, X-axis). Directionally prioritised genes are on the diagonal or closely below it (blue; P < 0.05 from DPM) while penalised genes are further below the diagonal (red). Gene P-values shown were not adjusted for multiple testing. **(C)** Enrichment map of pathways and processes representing the multi-omics phenotype of *IDH*-mutant gliomas. The network shows pathways as nodes that are connected by edges if the corresponding pathways share many genes. Groups of pathways lost in the directional analysis (pink background) or gained in the directional analysis (green background) are grouped on the right.
