# Supplementary material for: Directional integration and pathway enrichment analysis for multi-omics data
Source: Nat Commun. 2024 Jul 7;15:5690. doi: 10.1038/s41467-024-49986-4 (PMC11227559; doi:10.1038/s41467-024-49986-4)
Supplement: Supplementary file 3 — Description of Additional Supplementary Files [file 41467_2024_49986_MOESM3_ESM.pdf]

Description of Additional Supplementary Files

File Name: Supplementary Data 1

Description: Benchmarking of DPM. Simulated datasets of P-values and directional information were merged using DPM and the modified Strube's method.

File Name: Supplementary Data 2

Description: Differentially expressed genes in patient-derived GBM cells from *HOXA10-AS* lncRNA knockdown (KD) and overexpression (OE) experiments.

File Name: Supplementary Data 3

Description: Enriched pathways in *HOXA10-AS* KD and OE experiments identified using non-directional analysis.

File Name: Supplementary Data 4

Description: Enriched pathways in *HOXA10-AS* KD and OE experiments identified using directional analysis (DPM).

File Name: Supplementary Data 5

Description: Cancer samples with matching transcriptomics and proteomics data in the CPTAC and TCGA datasets.

File Name: Supplementary Data 6

Description: Associations of protein and transcript expression levels with patient overall survival (OS) in ovarian cancer.

File Name: Supplementary Data 7

Description: Enriched pathways with OS associations in ovarian cancer identified using non-directional analysis.

File Name: Supplementary Data 8

Description: Enriched pathways with OS associations in ovarian cancer identified using directional analysis (DPM).

File Name: Supplementary Data 9

Description: Differential protein and transcript expression, and DNA methylation of *IDH*-mutant gliomas.

File Name: Supplementary Data 10

Description: Enriched pathways in *IDH*-mutant gliomas identified using non-directional analysis.

File Name: Supplementary Data 11

Description: Enriched pathways in *IDH*-mutant gliomas identified using directional analysis (DPM).

File Name: Supplementary Data 12

Description: Directionally prioritised genes in *IDH*-mutant gliomas identified in the validation dataset.

File Name: Supplementary Data 13

Description: Enriched pathways in *IDH*-mutant gliomas identified using non-directional analysis in the validation dataset.

File Name: Supplementary Data 14

Description: Enriched pathways of *IDH*-mutant gliomas using directional analysis (DPM) in the validation dataset.
