# Supplementary material for: Directional integration and pathway enrichment analysis for multi-omics data
Source: Nat Commun. 2024 Jul 7;15:5690. doi: 10.1038/s41467-024-49986-4 (PMC11227559; doi:10.1038/s41467-024-49986-4)
Supplement: Supplementary file 18 — Reporting Summary [file 41467_2024_49986_MOESM18_ESM.pdf]

nature portfolio

Corresponding author(s): Jüri Reimand

Last updated by author(s): Jun 18, 2024

Reporting Summary

Nature Portfolio wishes to improve the reproducibility of the work that we publish. This form provides structure for consistency and transparency in reporting. For further information on Nature Portfolio policies, see our Editorial Policies and the Editorial Policy Checklist.

Statistics

For all statistical analyses, confirm that the following items are present in the figure legend, table legend, main text, or Methods section.

n/a	Confirmed	
☐	☒	The exact sample size (*n*) for each experimental group/condition, given as a discrete number and unit of measurement
☐	☒	A statement on whether measurements were taken from distinct samples or whether the same sample was measured repeatedly
☐	☒	The statistical test(s) used AND whether they are one- or two-sided<br>*Only common tests should be described solely by name; describe more complex techniques in the Methods section.*
☐	☒	A description of all covariates tested
☐	☒	A description of any assumptions or corrections, such as tests of normality and adjustment for multiple comparisons
☐	☒	A full description of the statistical parameters including central tendency (e.g. means) or other basic estimates (e.g. regression coefficient) AND variation (e.g. standard deviation) or associated estimates of uncertainty (e.g. confidence intervals)
☐	☒	For null hypothesis testing, the test statistic (e.g. *F*, *t*, *r*) with confidence intervals, effect sizes, degrees of freedom and *P* value noted<br>*Give P values as exact values whenever suitable.*
☒	☐	For Bayesian analysis, information on the choice of priors and Markov chain Monte Carlo settings
☒	☐	For hierarchical and complex designs, identification of the appropriate level for tests and full reporting of outcomes
☐	☒	Estimates of effect sizes (e.g. Cohen's *d*, Pearson's *r*), indicating how they were calculated

Our web collection on statistics for biologists contains articles on many of the points above.

Software and code

Policy information about availability of computer code

Data collection	The input datasets used in the study were downloaded from public repositories and supplementary tables of previous publications. We did not collect new data.
Data analysis	The DPM method is available as part of the ActivePathways R package in the CRAN repository (https://cran.r-project.org/web/packages/ActivePathways/) and on GitHub (https://github.com/reimandlab/ActivePathways). Custom scripts to prepare the input data and visualise the results as well as most input datasets are available on GitHub (https://github.com/reimandlab/DPM_publication_code). The source code of ActivePathways used in this study has been archived in Zenodo at DOI: 10.5281/zenodo.12118089.

For manuscripts utilizing custom algorithms or software that are central to the research but not yet described in published literature, software must be made available to editors and reviewers. We strongly encourage code deposition in a community repository (e.g. GitHub). See the Nature Portfolio guidelines for submitting code & software for further information.

Data

Policy information about availability of data

All manuscripts must include a data availability statement. This statement should provide the following information, where applicable:

- Accession codes, unique identifiers, or web links for publicly available datasets
- A description of any restrictions on data availability
- For clinical datasets or third party data, please ensure that the statement adheres to our policy

Datasets resulting from multi-omics data integration and pathway analyses are provided as Supplementary Data. Most input datasets required to generate and visualise the results are available on GitHub (https://github.com/reimandlab/DPM_publication_code). Input datasets from the GLASS project representing transcriptomics, methylation, and clinical profiles of gliomas are controlled-access and require additional approval by GLASS. GLASS datasets can be retrieved from the Synapse database at https://www.synapse.org (accession numbers: mRNA syn31121291; clinical: syn31121219; methylation: syn23594913).

Research involving human participants, their data, or biological material

Policy information about studies with human participants or human data. See also policy information about sex, gender (identity/presentation), and sexual orientation and race, ethnicity and racism.

Reporting on sex and gender	We did not report sex specific effects in the study. One section of the study involved ovarian cancer patients that were female. The analysis of IDH-mutant glioblastoma samples was limited to a small sample size that did not have sufficient statistical power to evaluate sex effects.
Reporting on race, ethnicity, or other socially relevant groupings	We did not report race, ethnicity or other socially relevant groupings in this study.
Population characteristics	For cancer samples from the CPTAC project, multi-omics data were compared with patient overall survival and patient age, patient sex, and tumor stage and/or grade were used as covariates. For multi-omics analyses of glioma samples, mutation status of IDH genes was used to compare glioma samples.
Recruitment	Our study did not involve patient recruitment as we used previously published datasets only. Informed consent was obtained from all human participants as part of previous studies.
Ethics oversight	Ethical review of the current data analysis project was granted by the University of Toronto Research Ethics Board under protocol no. 37521.

Note that full information on the approval of the study protocol must also be provided in the manuscript.

Field-specific reporting

Please select the one below that is the best fit for your research. If you are not sure, read the appropriate sections before making your selection.

☒ Life sciences ☐ Behavioural & social sciences ☐ Ecological, evolutionary & environmental sciences

For a reference copy of the document with all sections, see nature.com/documents/nr-reporting-summary-flat.pdf

Life sciences study design

All studies must disclose on these points even when the disclosure is negative.

Sample size	Sample size was not determined specifically as we used previously generated datasets.
Data exclusions	We excluded cancer samples with missing omics datasets to facilitate multi-omics data integration.
Replication	We repeated the multiomics integration and pathway enrichment analysis of IDH-mutant GBMs in an additional cohort of glioma samples and recovered a large fraction of similar biological processes and functional themes, providing evidence of replication.
Randomization	No randomization studies were performed as this project involved reanalysis of existing datasets.
Blinding	Blinding was not performed as this project involved reanalysis of existing datasets.

Reporting for specific materials, systems and methods

We require information from authors about some types of materials, experimental systems and methods used in many studies. Here, indicate whether each material, system or method listed is relevant to your study. If you are not sure if a list item applies to your research, read the appropriate section before selecting a response.

Materials & experimental systems

n/a	Involved in the study
☒	☐ Antibodies
☒	☐ Eukaryotic cell lines
☒	☐ Palaeontology and archaeology
☒	☐ Animals and other organisms
☐	☒ Clinical data
☒	☐ Dual use research of concern
☒	☐ Plants

Methods

n/a	Involved in the study
☒	☐ ChIP-seq
☒	☐ Flow cytometry
☒	☐ MRI-based neuroimaging

Clinical data

Policy information about clinical studies

All manuscripts should comply with the ICMJE guidelines for publication of clinical research and a completed CONSORT checklist must be included with all submissions.

Clinical trial registration	This study did not include clinical trials.
Study protocol	This study only used published and publicly available clinical datasets with basic clinical information from TCGA, CPTAC, and GLASS projects.
Data collection	Data were collected from public databases and publications.
Outcomes	Outcome data involved overall survival information that was collected from public databases. Survival analysis of multi-omics datasets included tumor grade and/or stage and patient age and sex as covariates. Multi-omics comparisons of glioma samples included mutation status of IDH genes to define the compared subsets of samples.

Plants

Seed stocks	NA - no plants were included in the study.
Novel plant genotypes	NA - no plants were included in the study.
Authentication	NA - no plants were included in the study.
